# Does age matter? - A MRI study on peritumoral edema in newly diagnosed primary glioblastoma

**DOI:** 10.1186/1471-2407-11-127

**Published:** 2011-04-12

**Authors:** Clemens Seidel, Nils Dörner, Matthias Osswald, Antje Wick, Michael Platten, Martin Bendszus, Wolfgang Wick

**Affiliations:** 1 Department of Neurooncology University Clinic Heidelberg, Im Neuenheimer Feld 400, D-69120 Heidelberg, Germany; 2 Department of Neuroradiology, University Clinic Heidelberg, Im Neuenheimer Feld 400, D-69120 Heidelberg, Germany

**Keywords:** age, brain tumor, glioblastoma, imaging, necrosis, vascular endothelial growth factor

## Abstract

**Background:**

Peritumoral edema is a characteristic feature of malignant glioma related to the extent of neovascularisation and to vascular endothelial growth factor (VEGF) expression.

The extent of peritumoral edema and VEGF expression may be prognostic for patients with glioblastoma. As older age is a negative prognostic marker and as VEGF expression is reported to be increased in primary glioblastoma of older patients, age-related differences in the extent of peritumoral edema have been assessed.

**Methods:**

In a retrospective, single-center study, preoperative magnetic resonance imaging (MRI) scans of steroid-naïve patients (n = 122) of all age groups were analysed. Patients with clinically suspected, radiologically likely or known evidence of secondary glioblastoma were not included.

Extent of brain edema was determined in a metric quantitative fashion and in a categorical fashion in relation to tumor size. Analysis was done group-wise related to age. Additionally, tumor size, degree of necrosis, superficial or deep location of tumor and anatomic localization in the brain were recorded.

**Results:**

The extent of peritumoral edema in patients >65 years (ys) was not different from the edema extent in patients ≤ 65 ys (p = 0.261). The same was true if age groups ≤ 55 ys and ≥ 70 ys were compared (p = 0.308). However, extent of necrosis (p = 0.023), deep tumor localization (p = 0.02) and frontal localisation (p = 0.016) of the tumor were associated with the extent of edema. Tumor size was not linearly correlated to edema extent (Pearson F = 0.094, p = 0.303) but correlated to degree of necrosis (F = 0.355, p < 0.001, Spearman-Rho) and depth of tumor (p < 0.001). In a multifactorial analysis of maximum edema with the uncorrelated factors age, regional location of tumor and degree of necrosis, only the extent of necrosis (p = 0.022) had a significant effect.

**Conclusion:**

Age at diagnosis does not determine degree of peritumoral edema, and tumor localization in the white matter is associated with greater extent of edema. The area of necrosis is reflective of volume of edema. In summary, the radiographic appearance of a glioblastoma at diagnosis does not reflect biology in the elderly patient.

## Background

Peritumoral edema is a characteristic feature of malignant glioma, related to the extent of neovascularisation and to vascular endothelial growth factor (VEGF) expression [1-3]. It is well recognized that VEGF is a major and potent mediator of blood brain barrier disturbance and a cause of peritumoral edema [4,5].

Some studies have reported a correlation between VEGF expression and extent of peritumoral edema [6,7]. Others show an association of extensive peritumoral edema on magnetic resonance imaging (MRI) with bad prognosis in patients with newly diagnosed glioblastoma [8-10].

Additionally, a recent study demonstrated that increased VEGF expression is more frequent in older patients with glioblastoma [11].

The aim of our study was to examine whether peritumoral edema is more pronounced in elderly patients with primary glioblastoma. We assessed whether increasing edema accounts for the well-known worse prognosis of glioblastoma with increasing age [12].

## Methods and Patient characteristics

### Methods

In this retrospective, single-center study, we analyzed preoperative MRI scans at first (suspected) diagnosis in two groups of steroid-naïve patients (≤ 65 ys and >65 ys) with primary glioblastoma. The patients were consecutively seen in our center between 2004 and 2010.

Patients with known or radiological evidence of secondary glioblastoma were excluded. Only 5/122 had areas suspicious for low-grade tumor but no clinical history of prior tumor manifestation. For all patients, preoperative MRI including native and contrast-enhanced T1-w and T2-w sequences were available. Analysis was done on digital images on a workstation (Leonardo, Siemens, Erlangen, Germany).

Necrosis and extent of edema and maximum tumor size were determined on axial contrast-enhanced T1- and T2-w MRI images, respectively. When edema extension was greater in the cranio-caudal direction than in the axial direction, coronal or sagittal images were used for edema determination.

To accurately quantify the local extent of maximum edema, the distance from the outer edge of maximum edema to the nearest point of contrast enhancing tumor border was measured in mm as described elsewhere [10]. Contrast-enhanced tumor was used to assess tumor size.

**Figure 1:**
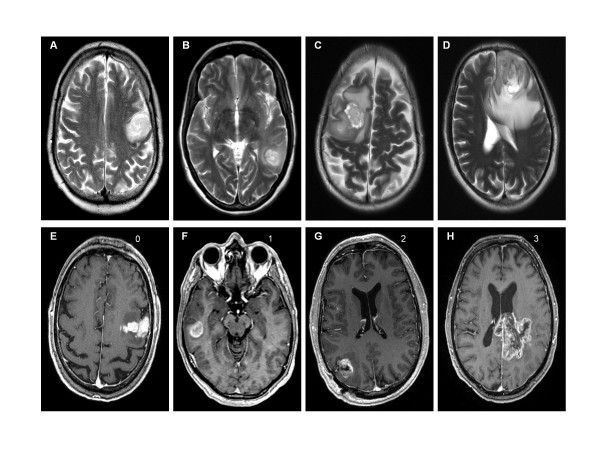
**Examples for two-dimensional measurement and scoring of edema (A-D) and necrosis (E-H)**. A-D, scoring of edema (t2-w -contrast MRI). E-H, scoring of degree of necrosis (t1-w +contrast MRI).

To describe the two-dimensional extent of edema in relation to tumor size a categorical scoring system was used, similar to what has been reported by others [9] (Table 1, Figure 1A-D). A standardized volumetric approach is not available.

**Table 1:** Grading system of edema and necrosis, (in analogy to [8])

Grade	Edema
0	No edema
1	Minimal edema
2	Edema approximately equal to tumor area
3	Major edema greater than tumor area
	
Grade	Necrosis
	
0	no necrosis
1	necrosis <25% of tumor area
2	necrosis 25-50% of tumor area
3	necrosis >50% of tumor area

To detect a 30% difference, 61 patients were included in each group. In a second analysis, to exclude an overlap of pathophysiological effects in patients aged 55-70 ys, different age limits were set (≤ 55, 55-69, and ≥ 70 ys).

As potential confounders, we assessed: *1) *largest tumor area (in mm^2^) on a single slice, *2) *superficial or deep localization in the brain (defined as being predominantly located in the grey matter for a superficial and deep white matter for a deep localization), *3) *degree of necrosis (hypointense region on T1-w in the centre of contrast-enhancing tumor, scored as in Table 1, Figure 1E-H) and *4) *regional location of tumor (frontal, pericentral, temporal, parietooccipital, basal ganglia). To avoid observer bias, radiological analysis was performed by an experienced neuro-oncologist (C.S.) and referenced by an independent neuroradiologist (N.D.). Both observers were blinded for clinical data including patient age.

### Patient characteristics

All group characteristics are shown in Table 2. In the primary data set (age cut-off 65 ys) 61 patients per group were included. The groups ≤ 55 ys, 55-69 ys and ≥ 70 ys comprised 42, 41 and 39 patients, respectively. There was no significant difference in mean tumor area (p = 0.706, t-test) or degree of necrosis (p = 0.173, Mann-U-Whitney) between the two groups with age limit 65 ys. The same applied to the three groups of patients with the age limits 55 ys and 70 ys (p = 0.140, ANOVA).

**Table 2:** Characteristics of n = 122 patients (patient age and morphological tumor parameters in MRI)

Age group	Age ≥ 65 ys	Age >65 ys	Age ≥ 55 ys	Age 55-69 ys	Age ≥ 70 ys
**Nr. of patients**	n = 61	n = 61	n = 42	n = 41	N = 39

**Age [years]**					
**Mean**	**51**	**72**	**46**	**64**	**75**
Standard dev.	8.91	4.64	7.5	4.3	3.7

**Maximum edema [mm]**					
**Mean**	**23.6**	**21**	**23.5**	**22.7**	**20.6**
Standard dev.	11.96	12.96	11	12.4	14.2

**Degree of edema**					
**Mean**	**1.75**	**1.43**	**1.67**	**1.78**	**1.31**
**Median**	2	1	1.5	2	1
Standard dev.	1.11	1.12	1.07	1.13	1.13

**Tumor area [mm**^**2**^**]**					
**Mean**	**1344**	**1285**	**1411**	**1271**	**1258**
Standard dev.	814	903	885	858	837

**Degree of necrosis [n (in %)]**					
**none**	1 (1.6%)	3 (4.9%)	1 (2.4%)	0	3 (7.7%)
**<25%**	4 (6.6%)	8 (13.1%)	2 (4.8%)	4 (9.8%)	6 (15.4%)
**25-50%**	13 (21.3%)	13 (21.3%)	11 (26,1%)	8 (19.5%)	7 (17.9%)
**>50%**	43 (70.5%)	37 (60.7%)	28 (66.7%)	29 (70.7%)	23 (59.0%)

**Tumor localisation [n (in %)]**					
***Depth:***					
Superficial	10 (16.4%)	9 (14.8%)	5 (11.9%)	7 (17.1%)	7 (17.9%)
Deep	51 (83.6%)	52 (85.2%)	37 (88.1%)	34 (82.9%)	32 (82.1%)
***Region of brain:***					
Frontal	22 (36.1%)	13 (21.3%)	16 (38.1%)	10 (24.4%)	9 (23.1%)
Temporal	15 (24.6%)	20 (32.8%)	11 (26.2%)	7 (17.1%)	9 (23.1%)
Central	14 (23.0%)	13 (21.3%)	7 (16.7%)	16 (39.0%)	12 (30.8%)
Parietooccipital	6 (9.8%)	13 (21.3%)	4 (9.5%)	7 (17.1%)	8 (20.5%)
Basal ganglia	3 (4.9%)	2 (3.3%)	3 (7.1%)	1 (2.4%)	1 (2.5%)
Other	1 (1.6%)	0	1 (2.4%)	0	0

## Results

### Do older patients with primary glioblastoma exhibit more peritumoral edema?

**Figure 2:**
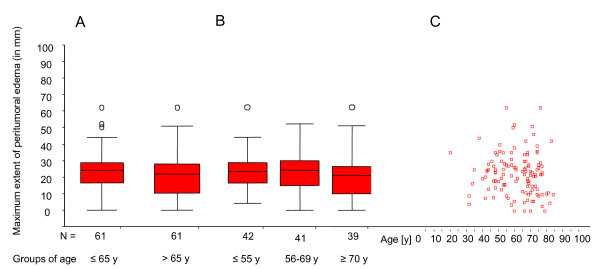
**Comparison of different age groups**.

Edema extent did not differ significantly between the age groups. This was consistently shown for the determination of the maximum extent of edema (p = 0.261, t-test, Figure 2A) and for the degree of edema as determined by categorical scoring in relation to tumor size. The latter showed a trend toward less edema (p = 0.106, Jonckheere-Terpstra) in patients >65 ys.

When patient groups ≤ 55 ys and ≥ 70 ys were compared, maximum extent of edema did not differ (p = 0.308, t-test, Figure 2B). Edema degree was lower in the group of older patients but did not reach statistical significance (p = 0.133, Jonckheere-Terpstra; data not shown).

As expected from the above, there was no correlation between age and maximum extent of edema (Pearson correlation coefficient: -0.076, p = 0.407, Figure 2C).

### Which factors do influence the extent of peritumoral edema?

#### Localisation of tumor

**Figure 3:**
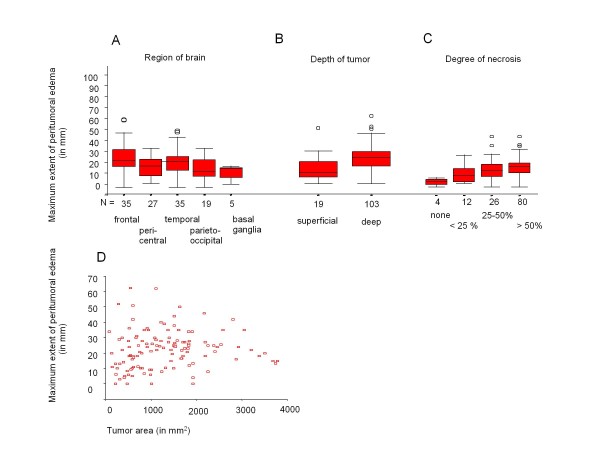
**Confounding factors**.

**Figure 4:**
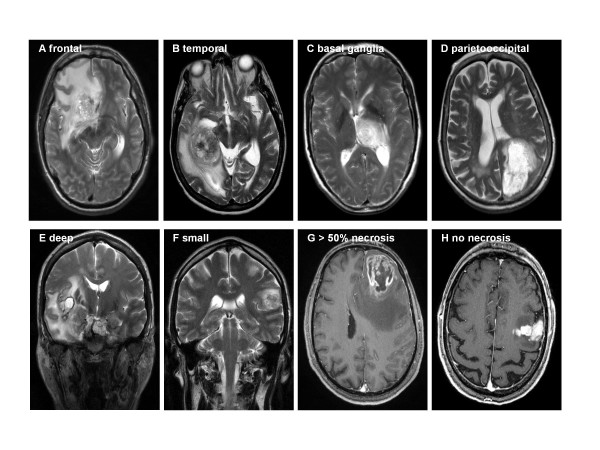
**Examples of different degrees of edema: at different localisations**. (A) frontal, B) temporal, C) basal ganglia, D) parietooccipital; E) superficial and F) deep tumor, G) >50% necrotic tumor, H) non-necrotic tumor

Interestingly, there are differences between different tumor localisations and the maximum peritumoral edema (p = 0.016; ANOVA, Figure 3*A) *as well as the degree of edema (p = 0.042, Krustal-Wallis Test, data not shown). The largest maximum edema was seen in frontal (n = 35) and temporal (n = 35) tumors whereas in other regions (pericentral [n = 27], parietooocipital [n = 19], basal ganglia [n = 5]) maximum tumor edema appeared to be less extensive. Examples are shown in Figure 4*(A frontal, B temporal, C basal ganglia, D parietooccipital)*. With Bonferroni corrections there is a strong trend for larger perifocal edema of frontal tumors compared to tumors in pericentral or parietoccipital regions (p = 0.054 and 0.057). There were no differences between tumors in the other regions.

Deeply located, mainly white matter tumors (n = 103) had a higher maximum edema (p = 0.02, t-test) and also a higher degree of edema (p = 0.006, Krustal-Wallis) than superficial, mainly grey matter tumors (n = 19) (Figure 3B). Some examples are shown in Figure 4E-F. Superficial tumors were significantly smaller than deep tumors (p < 0.001, t-test).

#### Degree of necrosis

Tumors with a higher degree of necrosis were found to have a higher maximum edema (p = 0.012, ANOVA) and a higher degree of edema (p = 0.023, Jonckheere-Terpstra) than tumors with less necrosis (Figure 3C). Some examples are shown in Figure 4G-H. After Bonferroni correction maximum edema only differed significantly between no and >50% necrosis (p = 0.029). No effect was seen for the edema index.

Degree of necrosis is positively correlated with tumor depth (F = 0.281, p = 0.002, *Spearman-Rho*) and tumor size (F = 0.355, p < 0.001, *Spearman-Rho*).

#### Tumor size

There was no correlation between tumor size and maximum extent of edema (F = 0.094, p = 0.303, Pearson). In a regression analysis a trend towards a quadratic regression of tumor area and extent of maximum edema (R^2 ^= 0.047, F = 2.56, p = 0.056, Figure 3D) was observed.

#### Multifactorial analysis

In a multifactorial analysis (general linear model) of the variable maximum edema with the uncorrelated factors age, regional location of tumor and degree of necrosis, only degree of necrosis had a significant effect (p = 0.022, Table 3). R^2 ^of this model was 0.794. If necrosis was replaced by depth of tumor or tumor size, only depth of tumor showed an effect (p = 0.016; p = 0.332).

**Table 3:** Multifactorial analysis of the variable Maximum edema extent

Factor	F-Value	p-Value
Age	0.724	0.820

Regional localisation of tumor	0.884	0.508

Degree of necrosis*	3.896	0.022

Depth of tumor*	6.373	0.017

* alternate factors

This result indicates that the degree of necrosis is the strongest independent factor influencing extent of edema.

## Discussion

As a principal finding of this analysis the extent of tumor edema in patients with primary glioblastoma is not age-related. We conclude that the bad prognosis of elderly patients compared to younger patients with primary glioblastoma [13,14] cannot be attributed to more perifocal edema.

Additionally, it has been suggested that VEGF expression, which is the major cause of brain tumor edema [15,16], may be higher in primary glioblastomas in older patients [11]. On a descriptive radiological level without VEGF level determination, this notion is not supported by the data of our study. In contrast to this, it has been shown for the VEGF antibody, bevacizumab, that older age may in fact be associated with a better response [11,17]. Therefore, careful analysis of older patients in current trials or even separate trials for patients > 65 years will most likely be rewarding.

Interestingly, large tumor size and extensive necrosis, which some authors linked to bad prognosis in the past [9,18] were not found to be more frequent in older patients.

In the multifactorial analysis, presence and extent of peritumoral edema in primary glioblastoma was associated only with depth of tumor or the extent of necrosis, implying that edema is the result of severe hypoxia. This may be regarded as morphological evidence for the pathophysiological link between hypoxia, hypoxia-inducible factor 1 alpha expression and VEGF-mediated genesis of peritumoral edema [19,20].

Additionally, in univariate analysis, extent of peritumoral edema differed between different regions of brain, possibly related to differences of structure and direction of white matter tracts. The structure and density of white matter is known to vary in different regions of brain, and edema spread tends to be influenced by the anatomy of white matter tracts [21,22]. Less dense white matter, e.g. in frontal association fibers, could facilitate edema spread, whereas denser commisural fibers, e.g. in the posterior corpus callosum, or projection fibers, e.g. in the corticospinal tract, may interfere with edema extension [23]. This effect might have contributed to our observation of accentuated edema in frontal white matter compared to less pronounced edema of tumors of the basal ganglia or the parietoccipital regions.

Tumor size did not appear to linearly correlate with extent of edema but a trend towards a quadratic relationship of tumor area to peritumoral edema existed. This may have been due in part to some very large tumors that involved most of the surrounding white matter and exhibited less edema because of the shortage of "edema substrate".

The factors tumor size, degree of necrosis and depth of tumor appeared to be partially correlated with each other. An interaction between these factors seems logical, as a larger glioblastoma might inherently have more extensive necrosis and a higher chance of involving deep white matter.

Our data are partially in conflict with results of others that found a correlation between increasing edema (in a three step scoring system, comparable to our score) and advanced age in 110 patients. Interestingly, a high proportion of the tumors analysed in this study showed areas of non-contrast enhancing tumor (65% in a group <50 ys, 35% in a group > 50 ys) [8]. In this study the presence of non-contrast enhancing tumor was associated with less edema. The contradicting results may reflect the differences in study populations. Due to the exclusion criteria, our study was much less likely to include secondary glioblastoma, which is known to be a genetically different entity with less VEGF expression, progressing slowly from non-enhancing tumor and only developing areas with necrosis and edema later in the course of disease [24-26].

In newly diagnosed "primary" tumors, Pope et al. assessed gene expression and reported that tumors with non-contrast enhancing parts, i.e. morphological signs of secondary glioblastoma, differ from tumors without non-contrast enhancing parts. Pro-angiogenic expression patterns including VEGF-overexpression were present in typical primary glioblastoma without non-contrast enhancing parts, whereas tumors with non-contrast enhancing areas overexpressed genes that were more suggestive of secondary glioblastoma [27].

The presence of genetic signatures of secondary glioblastoma such as mutated isocitrate dehydrogenase 1 is much higher in younger patients [28]. Thus, the notion that glioblastoma swith less peritumoral edema are more frequent in younger patients appears straightforward, reflecting the higher frequency of genetic patterns of secondary glioblastoma in this age group. The mean patient age in [8] is 54.9 years (CI 52.0-57.8), whereas in our study the mean patient age is 61.4 years (CI 59.06-63.68). Age composition of study groups appears crucial for evaluation of morphologic features of glioblastoma, as the percentage of tumors with clinical evidence or genetic signatures of secondary glioblastoma will influence the measured parameters, especially peritumoral edema.

## Conclusion

In conclusion, our study demonstrates that the extent of peritumoral edema in primary glioblastoma without relevant non-contrast enhancing tumor tissue is influenced by the degree of necrosis and the position of tumor in white matter. Age at diagnosis does not determine the degree of peritumoral edema. Tumor size, extent of necrosis and depth of tumor partially interact.

For daily clinical practice in older patients with glioblastoma, our data do not support enhanced steroid treatment or differential use of other anti-edema treatments, such as the antiangiogenic anti-VEGF(R) treatments [29], although older age in contrast to all other studies may not be a negative prognostic factor in anti-VEGF treatment [11,17]. However, it might be interesting to evaluate the differential effects of various anti-edema treatments based on tumor localisation or degree of necrosis.

## Abbreviations

VEGF: vascular endothelial growth factor; MRI: magnetic resonance imaging

## Competing interests

The authors declare that they have no competing interests.

## Authors' contributions

CS developed the study design, performed first MRI-analysis, statistical analysis and wrote the manuscript together with WW. ND performed reference MRI-analysis. MO participated in data collection and drafting of the manuscript.

AW and MP participated in study conduct and manuscript writing. MB coordinated radiological analysis. WW coordinated study design and statistical analysis and wrote the manuscript together with CS. All authors read and approved the final manuscript.

## Pre-publication history

The pre-publication history for this paper can be accessed here:

http://www.biomedcentral.com/1471-2407/11/127/prepub
